# Clinical characteristics and therapeutic response of immunoglobulin G4-related disease: a retrospective study of 127 Chinese patients

**DOI:** 10.1186/s13023-022-02404-8

**Published:** 2022-08-04

**Authors:** Wen An, Zhen Wu, Min Li, Haitian Yu, Xinyan Zhao, Xiaoming Wang, Yu Wang, Qianyi Wang, Weijia Duan, Yuanyuan Kong, Hong Ma, Xiaojuan Ou, Hong You, Yanying Liu, Peng Li, Ting Duan, Jidong Jia

**Affiliations:** 1 Liver Research Center, Beijing Friendship Hospital, Capital Medical University; Beijing Key Laboratory of Translational Medicine on Cirrhosis, National Clinical Research Center for Digestive Diseases, No.95 Yong-an Road, Xicheng District, Beijing, 100050 People’s Republic of China; 2 Center for Clinical Epidemiology and EBM, Beijing Friendship Hospital, Capital Medical University; Beijing Key Laboratory of Translational Medicine on Cirrhosis, National Clinical Research Center for Digestive Diseases, No.95 Yong-an Road, Xicheng District, Beijing, 100050 People’s Republic of China; 3 Department of Gastroenterology, Beijing Friendship Hospital, Capital Medical University; Beijing Key Laboratory of Translational Medicine on Cirrhosis, National Clinical Research Center for Digestive Diseases, No.95 Yong-an Road, Xicheng District, Beijing, 100050 People’s Republic of China; 4 Department of Rheumatology, Beijing Friendship Hospital, Capital Medical University; Beijing Key Laboratory of Translational Medicine on Cirrhosis, National Clinical Research Center for Digestive Diseases, No.95 Yong-an Road, Xicheng District, Beijing, 100050 People’s Republic of China

**Keywords:** IgG4-related disease, Clinical phenotype, Therapeutic response, Relapse, Predictive factors

## Abstract

**Background and aims:**

Immunoglobulin G4-related disease (IgG4-RD) is a multisystem fibroinflammatory condition. The aim of the present study was to characterize the clinical features and therapeutic response of patients with IgG4-RD and identify risk factors for disease relapse.

**Methods:**

We collected baseline data of eligible patients with IgG4-RD and analyzed clinical features by interview and review of medical records. The patients who received glucocorticoids (GC) therapy with at least 3 months follow-up were used to characterize the therapeutic response and identify risk factors for relapse.

**Result:**

Totally 127 IgG4-RD patients, including 92 males and 35 females, were enrolled in the present study. The median age of onset was 63.0 years, ranging from 23 to 86. The pancreas, bile duct and lymph nodes were the most frequently involved organs. The serum IgG4 level was elevated in 94.5% of the patients and was correlated with the number of organs involved. Patients classified as head and neck limited group were more likely to be female. Compared to Mikulicz syndrome and systemic involvement group, pancreato-hepatobiliary group had higher aminotransferase, alkaline phosphatase, gamma-glutamyl transpeptidase, bilirubin and lower IgG4 level. Mikulicz syndrome and systemic involvement group had the highest IgG4-RD RI score, IgG level. Among 92 patients who received medical therapy with at least 3 months follow-up, 76 received GC alone or in combination with immunomodulator (IM) and 16 patients did not take GC. 74 out of the 76 patients (97.3%) achieved remission, with 59 of them remained in remission and 15 of them relapsed. Whereas 16 patients did not take GC, among them, 6 patients achieved remission with one relapsed. On multivariate analysis, higher initial score of ACR/EULAR IgG4-RD Classification Criteria and GC withdrawal were independent predictors for relapse.

**Conclusion:**

Four phenotypes of IgG4-RD showed different demographic and serological features. GC + IM therapy was safe and effective and might protect patients from relapse. The independent risk factors of relapse were GC withdrawal and higher score of ACR/EULAR IgG4-RD Classification Criteria.

## Introduction

Immunoglobulin G4-related disease (IgG4-RD) is a multisystem fibroinflammatory condition characterized by male dominant, IgG4-positive plasma cell infiltration and elevated serum Immunoglobulin G4 (IgG4) levels [1]. Japanese researchers have estimated its incidence to be 0.28–1.08/100,000 inhabitants/year, with 336–1300 patients newly diagnosed per year [2]. Although the epidemiological study of IgG4-RD is rarely conducted in other part of the world, increasing number of cases has been reported from other countries including China in recent years [3].

Glucocorticoids are the first-line therapy for IgG4-RD and effective for most patients [4]. However, over 30% of patients had relapses during the tapering or after cessation of glucocorticoids (GC) [5, 6]. Till now, several risk factors for relapse in IgG4-RD patients have been identified [7–9]. However, whether adding immunomodulator agents to GC could reduce the relapse rate remains controversial [8, 10, 11]. Furthermore, the relationship between higher the American College of Rheumatology /the European League Against Rheumatism (ACR/EULAR) score [12] and relapse is still unknown.

Therefore, the aims of the study were to describe the clinical feature and identify risk factors for relapse in Chinese patients with IgG4-RD. We also explored the impact of different treatment strategies and higher scores of ACR/EULAR IgG4-RD Classification Criteria on the rate of relapse.

## Method

### Study population

This was a retrospective study of patients with IgG4-related diseases diagnosed between January 2014 and December 2020 at Beijing Friendship Hospital, Capital Medical University, China. This study was approved by the institutional ethics committee. (NO. YYXSSC-2021-097).

IgG4-related disease was diagnosed base on the Comprehensive Diagnostic Criteria for IgG4-related disease [13]: (1) characteristic diffused swelling or masses in single or multiple organs; (2) elevated serum IgG4 level (≥ 135 mg/dl); (3) histopathologic presentation showing: (1) marked lymphocyte and plasmacyte infiltration and fibrosis; (2) infiltration of IgG4 + plasma cells: ratio of IgG4 + /IgG + cells > 40% and > 10 IgG4 + plasma cells/HPF. Patients met (1) + (2) + (3), (1) + (3), or (1) + (2) were classified as definite, probable, or possible, respectively. Patients with pancreatic involvement who did not have pathological examination were diagnosed according to the International Consensus Diagnostic Criteria (ICDC) [14].

The inclusion criteria to the current study were: (1) diagnosis with Ig4-RD, and (2) with complete baseline data.

The exclusion criteria were: (1) with concomitant other autoimmune diseases, such as systemic lupus erythematosus, rheumatoid arthritis, ANCA associated systematic vasculitis; (2) complicated with malignant tumors.

### Baseline characteristics and follow-up information

The demographic, baseline data were collected by review of medical records: gender, age, complete blood count, erythrocyte sedimentation rate (ESR), biochemistry, serum immunoglobulin levels, IgG4 level, autoantibodies, imaging and histology. The serum fluorescence titers of anti-nuclear antibodies (ANA) were tested by the indirect immunofluorescence technique. The clinical phenotypes were classified based on organ involvement as reported by Wallace et al. group [15]: pancreato-hepatobiliary group, retroperitoneal fibrosis and/or aortitis group, head and neck-limited group and Mikulicz syndrome with systemic involvement group.

The follow-up information was obtained by interview in person or by telephone and review of medical records. Follow-up intervals were scheduled at month 1, 3 and every 3–6 months thereafter.

### Evaluation of therapeutic response

Therapeutic response to treatment with or without GC was evaluated by changes in IgG4-RD responder index (RI) [16] which was the sum of score referred to the degree of disease activity in all the organ sites, all graded on a 0–3 scale. Remission was defined as IgG4-RD RI declined ≥ 2 points from baseline. Disease relapse was defined as clinical symptoms recurred or imaging findings worsened after remission on glucocorticoids therapy. Elevated serum IgG4 level alone was not regarded as disease relapse.

### Statistical analyses

All statistical analyses were performed using SPSS version 26.0. Continuous variables were shown as median (interquartile range) or means with standard deviations. Categorical variables were displayed as counts and percentages. The Mann–Whitney U test was used for comparing non-normally distributed variables between different groups. The Chi-square test was applied to compare the status of categorical variables. Cox proportional hazards model was used to identify prognostic factors for relapse. The enter procedures were applied for the final model selection. The receiver operating characteristic (ROC) curve was used to determine the predictive ability of score of ACR/EULAR IgG4-RD Classification Criteria for relapse and the related cut-off values. Kaplan–Meier’s curve was used to assess relative contribution of variables to disease relapse. A two-sided probability *P* < 0.05 was considered statistically significant.

## Result

### Demographic and baseline clinical features of IgG4-RD

**Fig. 1:**
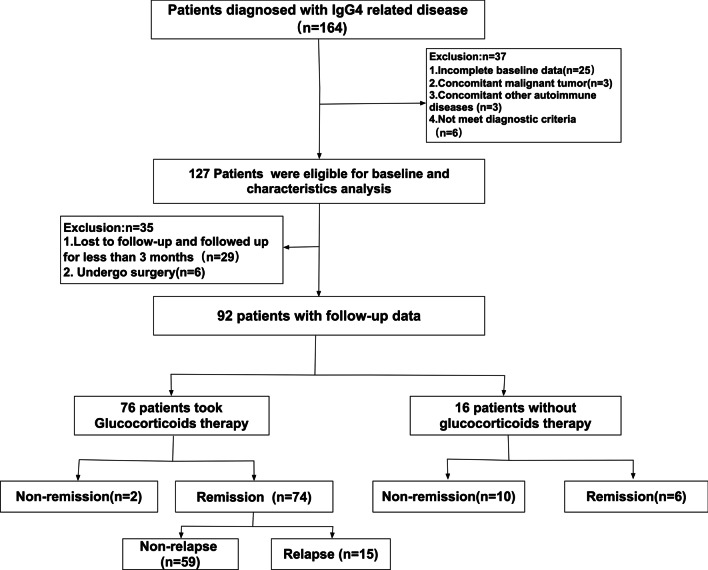
The flowchart for all patient enrollment

Totally 164 patients diagnosed with IgG4-related disease between January 2014 and December 2020 in our institute. 37 patients were excluded because of incomplete baseline data (n = 25), concomitant malignant tumor or other autoimmune diseases (n = 6) and not meet diagnostic criteria (n = 6). Finally, 127 IgG4-RD patients fulfilled the inclusion criteria and did not meet the exclusion criteria (Fig. 1). Among them, 92 were males and 35 were females (M: F = 2.62:1). The median age of onset was 63.0 years, ranging from 23 to 86.

According to the 2011 Comprehensive Diagnostic Criteria [13] for IgG4-RD, 15 (11.8%), 3 (2.3%) and 105 (82.6%) patients were diagnosed as definite, probable and possible IgG4-RD, respectively. Another 4 patients with normal serum IgG4 level were diagnosed as probable IgG4-RD according to the International Consensus Diagnostic Criteria. We used 2019 ACR/ EULAR Classification Criteria for IgG4-Related Disease as a tool to grade all patients and found that 104 patients (82%) fulfilled the 2019 ACR/ EULAR Classification Criteria for IgG4-Related Disease. The median score was 29 (IQR:22–35).

**Fig. 2:**
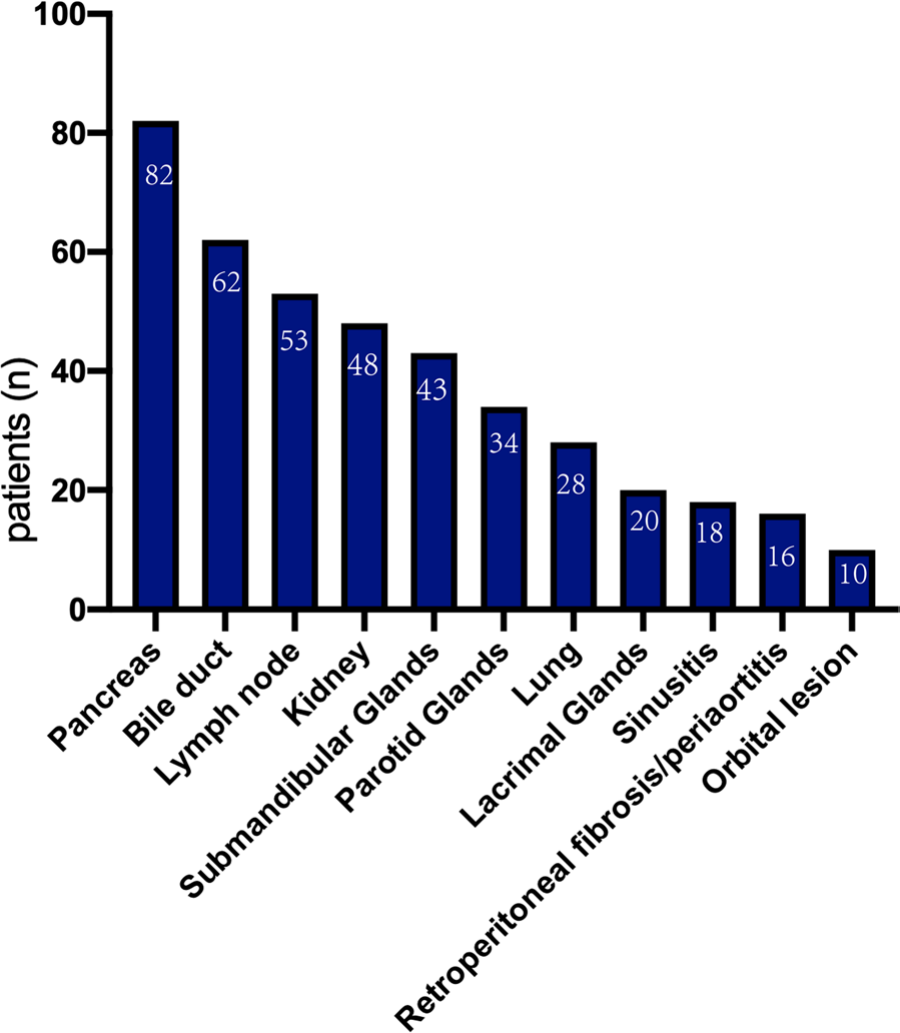
Organ involvement in 127 patients with IgG4-related disease

In our cohort, 25 patients (19.7%) showed single organ involvement, 87 patients (68.5%) showed 2–4 organs involvement, and 15 patients (8.6%) showed more than 5 organs involvement. Patients with 5 or over organ involvement group had higher serum IgG and IgG4 level than those for patients with less 5 organs involvement group (all *P* < 0.05). The most commonly involved organs were pancreas (n = 82,64.5%), bile duct (n = 62,48.8%), lymph nodes (n = 53,41.7%), and kidney (n = 48, 37.8%). (Fig. 2).

The most common clinical presentations of IgG4-RD were abdominal pain (34.0%), followed by jaundice (30.7%), and glands swelling (21.5%).

Table 1 showed the baseline laboratory findings for IgG4-RD patients. The median baseline serum levels of IgG level and IgG4 were elevated in this cohort (1775 g/L, 9.8 g/L), with 70 patients (70/120) having increased IgG level and 120 patients (94.5%) having increased IgG4 level. Low titer ANA was detected in 66 patients (66/76), none of whom were positive for anti-extractable nuclear antibodies.

**Table 1:** Demographics and serological features for 127 IgG4-related disease patients divided by different clinical phenotype

Characteristic^a^	All patients (n = 127)	Pancreato-hepato-biliary (n = 59)	RPF/Aorta (n = 6)	Head and neck-limited (n = 15)	Mikulicz and systemic (n = 47)	*P* value
Male gender, n (%)	92 (72.4%)	47 (79.7%)	6 (100%)	7 (46.7%)	32 (68.1%)	0.03
Age at onset (years)	63 (55–69)	64.0 (55.0–69.0)	61.0 (55.8–65.5)	62.0 (54.0–72.0)	63.0 (56.0–70.0)	0.9
WBC (10^9^/L)	5.7 (4.5–7.1)	5.5 (4.5–6.9)	9.6 (6.6–11.0)	6.1 (4.6–7.4)	5.5 (4.2–7.3)	0.02
Eosinophils (%)	4.4 (2.1–7.5)	4.5 (2.0–7.6)	3.3 (1.8–4.3)	4.3 (1.7–8.3)	4.5 (2.6–7.7)	0.7
ALT (U/L)	33.5 (15.0–134.8)	90 (32.0–175.0)	17.0 (12.8–35.3)	17 (14.0–24.5)	20.0 (12.8–83.3)	0.00
AST (U/L)	31.9 (19.8–91.3)	71.4 (26.0–130.5)	16.8 (15.3–22.2)	21.6 (18.8–25.7)	25.0 (18.3–81.5)	0.00
ALP (U/L)	102 (22.0–525.0)	316.5 (159.5–555.8)	98.0 (78.5–103.0)	76.0 (66.5–93.0)	79.0 (66.0–283.5)	0.00
GGT (U/L)	140.0 (74.0–392.0)	373.0 (70.0–817.0)	45.5 (32.3–88.8)	23.0 (17.0–31.0)	32.0 (15.0–236.0)	0.00
TBIL (umol/L)	35.9 (32.3–39.0)	53.9 (15.9–148.1)	9.8 (8.5–12.3)	10.8 (8.4–13.1)	14.5 (9.8–32.2)	0.00
DBIL (umol/L)	34.2 (30.3–40.6)	27.0 (4.4–98.0)	1.9 (1.5–2.7)	1.8 (1.5–2.3)	3.3 (1.9–12.9)	0.00
ALB (g/L)	18.2 (10.8–70.6)	35.5 (32.2–38.4)	38.2 (35.4–41.5)	38.6 (36.8–41.0)	35.7 (30.5–39.2)	0.03
GLO (g/L)	5.5 (2.1–37.0)	32.2 (29.5–37.7)	34.1 (29.7–40.0)	32.6 (30.0–42.8)	37.0 (32.3–48.6)	0.01
Cr (umol/L)	69.7 (58.1–84.2)	69.2 (54.8–77.5)	110.1 (79.8–140.1)	74.4 (62.5–91.0)	69.0 (56.8–89.5)	0.01
IgG (g/L)	1775 (1527–2357)	1680.0 (1475.0–2065.0)	1540 (1315.0–1870.0)	1545.0 (1240.0–2227.5)	2020.0 (1680.0–2850.0)	0.01
IgG4 (g/L)	9.8 (3.9–15.2)	10.3 (0.54–53.7)	3.0 (1.7–6.8)	8.2 (3.9–20.6)	13.0 (4.5–22.1)	0.01
IgG4-RD RI	8.0 (6.0–10.0)	6.0 (6.0–8.0)	10.0 (8.0–17.0)	6.0 (4.0–8.0)	10.0 (6.0–14.0)	0.00

RPF, retroperitoneal fibrosis; WBC, white blood cell; ALT, alanine aminotransferase; AST, aspartate aminotransferase; ALP, alkaline phosphatase; GGT, glutamyl transpeptidase; TBIL, total bilirubin; DBIL, direct bilirubin; ALB, albumin; GLO, globulin; CR, Creatinine; IgG, immunoglobulin G; IgG4, immunoglobulin G4; IgG4-RD RI, IgG4-Related Disease Responder Index

^a^All continuous variables were shown as median (Interquartile range)

The imaging feature of patients with IgG4-RD demonstrated organ enlargement of involved tissues. Local or diffuse pancreatic gland enlargement can be seen in 71 patients. Diffuse or segmental narrowing of bile duct and thickening of bile duct wall were seen in 45 patients. Lacrimal and salivary gland enlargement was commonly observed in 60 patients. Bilateral renal cortex low-density areas of the kidney were found in 10 patients.

Histopathological examinations were conducted in 55 patients, including biopsies of pancreas (n = 19), submandibular glands (n = 10), labial gland (n = 9), neck tumor (n = 4), lymph node (n = 3), kidney (n = 2), liver (n = 2), parotid glands (n = 1) and others (n = 5).

Among them, 18 patients (31.1%) fulfilled the pathological diagnostic criteria of IgG4-RD.

The characteristic histopathological findings of IgG4-related disease were dense lymphocytic infiltration and storiform fibrosis. In our cohort, the most common manifestation was lymphocytic and plasma cell infiltration (44.6%). Storiform fibrosis was observed in 6 patients (10.7%).

### Features of different clinical phenotypes of IgG4-RD

The clinical phenotypes were classified based on organ involvement as reported by Wallace et al. [15]. In our cohort, 59, 6, 15, and 47 patients belonged to the pancreato-hepatobiliary group, retroperitoneal fibrosis and/or aortitis group, head and neck-limited group and Mikulicz syndrome with systemic involvement group, respectively. (Table 1). Patients from head and neck limited group were more likely to be female (53.3%) than patients in the other groups.

We chose Mikulicz and systemic group as reference and compared other two groups separately with Mikulicz and systemic group. Compared to Mikulicz syndrome and systemic involvement group, pancreato-hepatobiliary group had higher aminotransferase, alkaline phosphatase (ALP), gamma-glutamyl transpeptidase (GGT), bilirubin and lower IgG4 level. Mikulicz syndrome and systemic involvement group had the highest IgG-RI score, IgG level.

## Therapeutic response in patients with more than 3-months follow-up

Out of the 127 patients, 29 patients followed up for less than 3 months, 6 patients received surgical treatment. Finally, 92 patients did not undergo surgery and had at least 3 months follow-up, with a median follow-up period of 17 months (IQR: 8–33 months).

Among 76 GC monotherapy or GC + IM therapy patients, 74 patients (97.3%) achieved remission, with 59 of them remained in remission and 15 of them relapsed. The median dose of prednisolone was 40 mg/d (IQR:30–40) and the median time was 11 months (IQR:6–26). The Mann–Whitney U test showed no statistical difference in baseline data between relapse and non-relapse patients.

Among these GC treated patients, steroid-induced necrosis of femoral head was observed in one, and poor glycemic control was reported in 7 patients. Gastrointestinal side effect, edema and poor blood pressure control occurred in one, two and three patients, respectively, which were regarded as steroid-related. GC and GC + Immunomodulator (IM) groups showed no statistical difference in side effects.

Another 16 patients did not take GC therapy. Among them, 6 patients achieved remission with one relapsed. Table 2 showed the involvement organ, treatment regimens and therapeutic response of the 16 patients.

**Table 2:** Clinical features, management and outcome of patients without GC therapy

Number	Gender	Involvement organ	Treatment regimen	Therapeutic response	Follow-up time (month)
1	M	Pancreas, Bile duct	Pancreatin Enteric-coated capsules	Non-remission	22
2	F	Pancreas, Submandibular gland, Lung	Hydroxychloroquine	Non-remission	11
3	M	Pancreas, Bile duct, Kidney, Submandibular gland	None	Non-remission	43
4	M	Submandibular gland, Pancreas, Lacrimal gland, Retroperitoneal fibrosis	None	Non-remission	25
5	M	Pancreas, Kidney	Pancreatin Enteric-coated capsules	Non-remission	13
6	M	Pancreas, Kidney, Lymph nodes	Tamoxifen	Non-remission	18
7	M	Pancreas, Bile duct	EMBE, UDCA	Non-remission	3
8	F	Pancreas	None	Non-remission	9
9	M	Pancreas, kidney	None	Non-remission	17
10	F	Pancreas, Bile duct	EMBE	Non-remission	19
11	M	Pancreas, Kidney	None	Remission	3
12	F	Pancreas	None	Remission	15
13	M	Pancreas, Bile duct	Pancreatin Enteric-coated capsules, UDCA	Remission	61
14	F	Pancreas, Bile duct	Pancreatin Enteric-coated capsules	Remission	13
15	M	Pancreas, Retroperitoneal fibrosis	None	Remission	15
16	F	Submandibular gland, Parotid gland	TwHF	Relapse	33

M, male; F, female; EMBE, endoscopic metal biliary endoprosthesis; UDCA, ursodeoxycholic acid; TwHF, Tripterygium Wilfordii Hook F

### Comparison of cumulative relapse rate in patients with different treatment regimen

There were no statistical significant differences between the remission and relapse groups in baseline laboratory findings (Table 3). Among 74 GC treated response patients, 51 patients received GC monotherapy (68.9%) and 23 patients (31.1%) received GC and IM combination therapy. The types of immunomodulators included methotrexate (n = 9), cyclophosphamide (n = 5), FK506 (n = 4), azathioprine (n = 2), leflunomide (n = 2) and mycophenolate mofetil (n = 1).

The Kaplan–Meier curve showed that the cumulative relapse rate in the GC monotherapy group was higher than that in the GC + IM therapy group (Log rank test, *P* = 0.01). There were no statistical significant differences between the GC monotherapy and GC + IM combination therapy groups in demographic features and baseline laboratory findings and initial GC dose.

**Table 3:** The demographics and baseline features for relapse and non-relapse patients

Characteristic^a^	Total follow-up patients (n = 74)	Remission patients (n = 59)	Relapse patients (n = 15)	*P* value
Male gender, n (%)	54 (73.0%)	40 (67.8%)	14 (93.3%)	0.05
Age at onset (years)	60.2 (23–79)	63 (56–68)	64 (52–66)	0.3
White blood cell (10^9^/L)	5.8 (1.0–12.2)	5.3 (4.2–7.3)	5.5 (4.7–7.3)	0.7
Eosinophils (%)	5.5 (0.07–19.6)	4.6 (2.25–7.65)	4.3 (2.7–6.5)	0.4
IgG (g/L)	2189.7 (1010.0–5590.0)	1795.0 (1570.0–2357.0)	1970 (1730–2605)	0.3
IgA (g/L)	190.0 (56.1–432.0)	187.0 (116.5–254.0)	206.5 (109.0–257.8)	0.8
IgM (g/L)	77.1 (20.1–227.0)	69.9 (43.6–101.5)	68.0 (40.1–87.0)	0.8
IgG4 (g/L)	15.6 (0.54–73.2)	10.9 (4.1–17.7)	13.2 (5.7–20.6)	0.3
IgG4-RD RI	10.0 (2–36)	8 (6–12)	8 (6–14)	0.2
Pancreato-hepato-biliary (n)	30	23	7	–^b^
RPF/Aorta (n)	2	2	0	–^b^
Head and neck-limited (n)	8	8	0	–^b^
Mikulicz and systemic (n)	34	26	8	–^b^

IgG, immunoglobulin G; IgA, immunoglobulin A; IgM, immunoglobulin M; IgG4, immunoglobulin G4; IgG4-RD RI, IgG4-Related Disease Responder Index; RPF, retroperitoneal fibrosis

^a^All continuous variables were shown as median (Interquartile range)

^b^Because of smaller sample size, we did not use the Chi-square test to compare those items between two groups

**Fig. 3:**
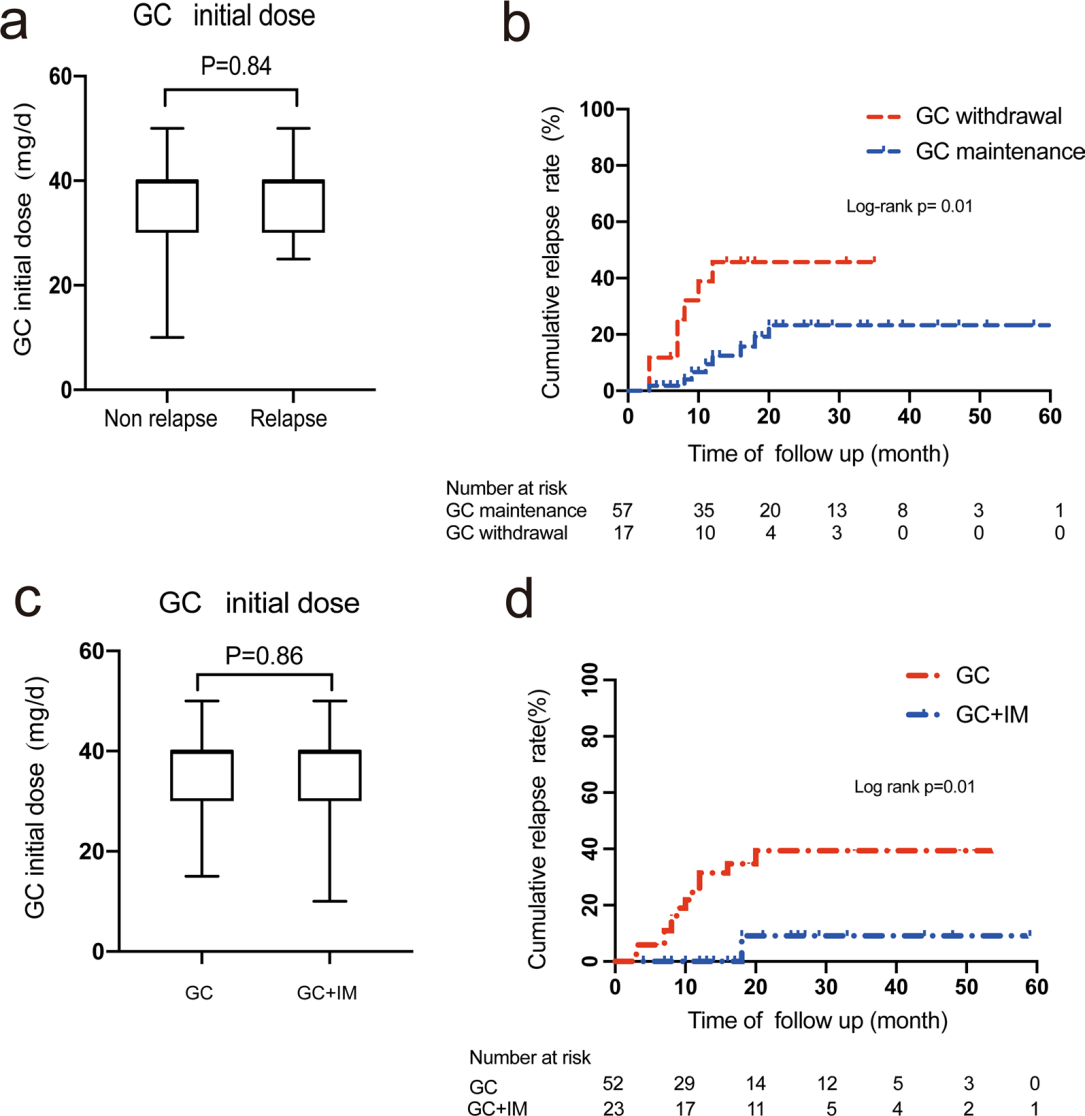
Analysis for 74 response to therapy patients in this study. **a** Initial prednisone dose for non relapse and relapse patients. **b** The cumulative relapse rate of GC maintenance and withdrawal groups. **c** Initial prednisone dose for GC and GC + IM patients. **d** The cumulative relapse rate of GC and GC + IM groups

In the maintenance treatment stage, 7 out of 17 GC withdrawal patients, (41.2%) suffered from relapse. The cumulative relapse rate was significantly higher for GC withdrawal patients (Log rank: *P* = 0.01). (Fig. 3).

### Comparison of cumulative relapse rate in patients with different score of ACR/EULAR IgG4-RD Classification Criteria level

**Fig. 4:**
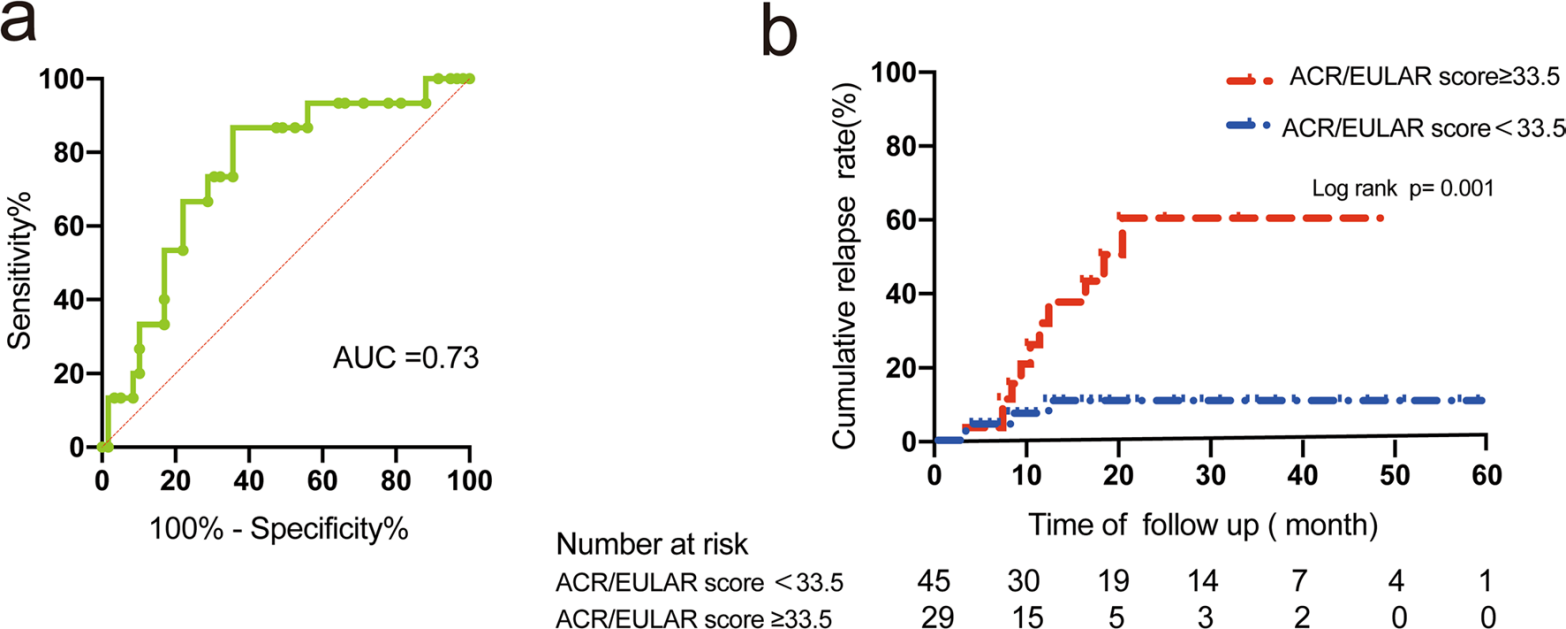
The relapse of patients with higher Score of ACR/EULAR IgG4-RD Classification Criteria. **a** The receiver operating characteristic (ROC) curve of Score of ACR/EULAR IgG4-RD Classification Criteria for relapse and the related cut-off values. **b** Comparison of the cumulative relapse rates of GC monotherapy and GC + IM therapy during the GC tapering stage

Cox regression analysis demonstrated that higher initial scores of ACR/EULAR IgG4-RD Classification Criteria were associated with relapse in patients with IgG4-RD (Table 4). The ROC curve showed that the maximum score cutoff set by using Youden’s index was 33.5 (sensitivity 73.3%, specificity 69.5%). The Kaplan–Meier plotting and log-rank test demonstrated that the score of ACR/EULAR IgG4-RD Classification Criteria ≥ 33.5 group had higher cumulative rate (Log rank: *P* = 0.01). (Fig. 4).

**Table 4:** Univariate and multivariate Cox regression of predictive factors for relapse in IgG4-RD

Variable	Univariate analysis			Multivariate analysis		
	*P* value	Odds ratio	95% CI	*P* value	Odds ratio	95% CI
*Demographics*						
Male, n (%)	0.10	5.29	0.69–40.32			
Age at onset, years	0.64	0.93	0.94–1.04			
Number of involved organs (≥ 5)	0.04	3.02	1.07–8.53			
Lacrimal gland	0.14	1.54	0.86–2.73			
Parotid	0.91	1.03	0.60–1.76			
Submandibular gland	0.79	0.93	0.56–1.56			
Lung	0.18	0.50	0.18–1.38			
Retroperitoneal fibrosis	0.37	1.34	0.71–2.52			
Pancreas	0.07	4.02	0.90–17.87			
Bile duct	0.07	1.65	0.96–2.85			
Lymph node	0.34	0.78	0.46–1.30			
Kidney	0.34	1.67	0.58–4.83			
IgG4, median (IQR), g/l	0.08	1.03	1.00–1.06	0.65	1.01	0.97–1.05
Serum IgG4 level ≥ 5 ULN	0.15	2.50	0.70–8.88			
IgG4-RD RI scores	0.04	1.06	1.00–1.12	0.69	0.98	0.91–1.07
Score of ACR/EULAR IgG4-RD Classification Criteria	0.004	1.07	1.02–1.11	0.02	1.10	1.02–1.19
Score of ACR/EULAR IgG4-RD Classification Criteria ≥ 20	0.55	0.54	0.07–4.08			
GC + IM therapy n (%)	0.04	0.12	0.02–0.93	0.06	0.14	0.02–1.06
Complete GC withdrawal	0.02	3.35	1.21–9.29	0.01	4.07	1.33–12.51

IgG4, immunoglobulin G4; ACR/EULAR, American College of Rheumatology/European League Against Rheumatism; GC, glucocorticoids

### Predictors of IgG4-RD relapse

Univariate analysis revealed that higher IgG4-RD RI scores at the baseline, the higher initial score of ACR/EULAR IgG4-RD Classification Criteria, over five organs involvement, GC-monotherapy and GC withdrawal were associated with relapse. Multivariate analysis using a Cox regression analysis further clarified that the higher initial score of ACR/EULAR IgG4-RD Classification Criteria, GC monotherapy and GC withdrawal were significant independent predictors of relapse in patients with IgG4-RD. (Table 4).

## Discussion

Our findings validated that four clinical phenotypes of IgG4-RD patients shared different demographic and serological features. As for therapeutic response, most patient achieved remission with GC monotherapy or GC + IMtherapy. GC withdrawal and higher score of ACR/EULAR IgG4-RD Classification Criteria were independent predictors for relapse.

In our cohort, the median age of onset in our cohort was 63 years old, and the ratio of male to female was 2.62:1, which was basically consistent with previous studies by other Chinese authors (median age:53.1, M:F = 2.3:1) [17]. The most common affected organ was pancreas which was different from some research [8, 17]. This difference may attribute to the fact that our center has advantages of subject in gastroenterology.

Patients with multiple organ involvement had higher level of baseline serum IgG and IgG4. It suggests that we should consider the possibility of multiple organ involvement if we observe a patient with a significant increase of IgG4 or IgG4.

Although there was no statistical difference in gender of clinical phenotypes. We observed that patients from head and neck limited group were more likely to be female (53.3%). Mikulicz syndrome with systemic involvement group had the highest serum IgG and IgG4-RD RI. These findings were in line with an Italian cohort and two international cross-sectional cohorts [15, 18]. Our study also validated that some demographic and serological features were quite different between each clinical phenotype [15]. It may provide physicians a new insight into this clinical classification of IgG4-RD.

Our result demonstrated that 97.4% patients (n = 74) response to GC therapy, only 2 patients did not achieve remission. 6 patients with milder degree of pancreas enlargement or head and neck-limited involvement achieved remission without GC treatment and the rest of 10 patients who did not take GC therapy were failed to achieve remission. It is widely accepted that glucocorticoids are the treatment of choice for IgG4-RD and effective for most patients [4]. Several studies have reported that patients with IgG4-RD who are not treated with GC are less likely to achieve remission [19, 20]. Our finding indicated that the majority of IgG4-RD patients with moderate-severe and/or multiple organ involvement may not benefit from the “Watchful waiting” strategy [4].

In this study, the relapse rate was 20.3% which was consistent with some researches [8, 9]. Cox regression showed that GC withdrawal, GC monotherapy and higher score of ACR/EULAR IgG4-RD Classification Criteria were independent prognostic factors for relapse. It is accepted that GC withdrawal is associated with disease relapse [9, 21, 22, 25]. In our cohort, 7 patients relapsed within median 4.3 months after glucocorticoids withdrawal. (OR 3.189, 95% CI 1.571–6.474, *P* = 0.001).

So far, the role of combination therapy in relapse still remains controversial. Several studies have reported that combination therapy associated with a lower relapse rate [9, 11, 23, 26]. Our study also revealed that patients who took GC + IM therapy were less likely to relapse than the GC monotherapy group. The result implied that combination therapy was safe and effective and might protect patients from relapse. On the contrary, a recent cohort showed that GC + IM therapy was not the risk factor of relapse [8]. This doubt may attribute to different types and maintenance periods of IM between medical centers. Standardized immunomodulator agent treatment is urgently needed for IgG4-RD patients in the future.

Our multivariate analysis showed that the higher score of ACR/EULAR IgG4-RD Classification Criteria was also associated with disease relapse. It seems that patients with higher scores of ACR/EULAR IgG4-RD Classification Criteria are likely to relapse and should own more attention. Score of ACR/EULAR IgG4-RD Classification Criteria was calculated as the sum of several weighted criteria including clinical findings, serological results, radiological assessments and pathological interpretations [12]. Higher score implied patients may have more organ involvement, higher serum IgG4 level or baseline IgG4-RD RI score. Some of those components (more organ involvement, higher serum IgG4 level) were reported as the risk relapse factors [9, 24, 27]. It may explain the relationship between higher score of ACR/EULAR IgG4-RD Classification Criteria and relapse.

Our study has several limitations. Firstly, this is a single-center and retrospective study. Large sample size and long duration follow-up studies are required to define the prognosis of the condition. Secondly, our cohort has a different spectrum of organ involvement; therefore, a multicenter study might help us better recognize the disease.

In conclusion, GC withdrawal was the risk factor of relapse. GC + IM therapy was safe and effective and might protect patients from relapse. For the first time, this study described that higher scores of ACR/EULAR IgG4-RD Classification Criteria may associated with relapse.

## Data Availability

The datasets used and/or analyzed during the current study are available from the corresponding author on reasonable request.
